# Randomised *in situ* clinical trial investigating self-assembling peptide matrix P11-4 in the prevention of artificial caries lesions

**DOI:** 10.1038/s41598-018-36536-4

**Published:** 2019-01-22

**Authors:** A. Jablonski-Momeni, H. Korbmacher-Steiner, M. Heinzel-Gutenbrunner, B. Jablonski, W. Jaquet, P. Bottenberg

**Affiliations:** 1 Philipps University of Marburg, Dental School, Department of Orthodontics, Marburg, Germany; 2 MH Statistics Consulting, Marburg, Germany; 3 Dental Practice, Lollar, Germany; 4 Vrije Universiteit Brussel, Department of Oral Health Sciences ORHE, Faculty of Medicine and Pharmacy, Brussel, Belgium; 5 Vrije Universiteit Brussel, Department of Educational Science EDWE-LOCI, Faculty of Psychology and Educational Sciences, Brussel, Belgium; 6 University of Ghent, Department of Periodontology and Oral Implantology, Dental School, Faculty of Medicine and Health Sciences, Ghent, Belgium

## Abstract

The aim was to investigate the ability of self-assembling Peptide P11-4 Matrix (SAPM) to remineralize artificial initial caries lesions compared to the use of fluoride varnish. Volunteers were recruited for this randomised, cross-over *in situ* trial. Bovine specimens, half including orthodontic brackets, were recessed on the buccal aspects of mandibular appliances. Specimens included internal sound enamel control, a demineralised control and a part exposed during the *in situ* phase. Each phase lasted four weeks, followed by a one-week washout. Treatment groups were: A: negative control, no treatment,B: positive control, 22,600 ppm fluoride varnish,C: test group, 1,000 ppm SAPM. Laser fluorescence values (LF) were measured before/after demineralisation, and after the *in situ* period. Micro-CT analysis was used to assess mineral changes within the specimens after the *in situ* phase. In specimens without brackets, ΔLF values after *in situ* phase were: A: +5.28, B: +0.85, C: −2.89. Corresponding ΔLF for specimens with brackets were: A: +5.77, B: +1.30, C: −3.15. LF-values between groups significantly differed from each other (p < 0.0001) after the *in situ* phase. Micro-CT analysis yielded no significant difference among groups for specimens without brackets. For specimens with brackets, the test group showed significantly more remineralisation than the negative (p = 0.01) and positive control (p = 0.003). Within the limitations of the study, SAPM showed prevention of caries and remineralisation of enamel around orthodontic brackets.

## Introduction

Caries is the most common disease in the world as reported by the Global Burden of Disease Study^1^. As caries progression is initiated by an imbalance of the remineralisation-demineralisation equilibrium favouring demineralisation^2–4^, any prevention should aim at shifting the equilibrium back towards remineralisation. This will lead to arrest of progression or even to regression of caries lesions^5,6^.

Recently a number of caries risk groups have been identified and caries risk assessment has become standard practice^7^. Persons wearing orthodontic appliances belong to one of the high risk groups^8^. The presence of fixed appliances reduces the efficacy of patient’s oral hygiene measures leading to oral and systemic health issues^9^. After bracket removal, white spot lesions may be present. These early lesions are known to undergo partial remission and/or inactivation of caries in the time following bracket removal as the cause of the caries, the plaque around the brackets, is no longer present^10^. Yet caries has often progressed to a cavity, and thus remission is no longer possible and restoration is the only treatment option^8^.

In order to prevent formation of white spot lesions, high-risk patients are often subject to a strict oral hygiene regimen, including application of products containing fluoride and calcium phosphate^11,12^. Fluoride has been shown to effectively prevent dental caries^13^. Yet recent studies have shown the limitations of fluoride when caries lesions have already progressed to the clinically manifest white spot stadium, leading at best to an arrest of the lesion’s activity^14,15^. Regardless, fluoride is the gold standard for caries prevention, meaning that any new approach should be a complement to fluoride in preventing and arresting caries more effectively and possibly lead to a regression of the early lesion. Nevertheless, products should be suitable for home and office use in order to promote compliance in high-risk patients.

In recent years biomimetic remineralisation strategies have been explored^16^. Many are modelled on the Amelogenin mode of action, which controls hydroxyapatite formation in enamel^17^. The biomimetic strategies aim at mineralisation of the subsurface caries lesion leading to additional mineralisation, whereas fluoride acts mainly in the superficial mineral layer of the caries lesion^18^.

The biomimetic strategies for the regeneration of enamel explored to date are Amelogenin itself, Amelogenin fragments (e.g. LRAP)^19,20^, or Amelogenin in combination with chitosan^17^. Neither has yet been incorporated into a commercial product, possibly due to the long peptide chains and the resulting production costs. An alternative that has been investigated in a number of clinical and *in vitro* studies is the self-assembling peptide P11-4. This peptide was shown to be able to diffuse into the subsurface lesion in monomeric form and there to undergo self-assembly into fibres promoting hydroxyapatite formation on the fibre surface^21,22^. The mechanism of action has been described in detail, and the superior remineralisation compared to placebo or fluoride varnish has been demonstrated^21–25^.

If applied on undemineralized enamel surfaces, fully self-assembled fibrillar P11-4 (i.e. self-assembling peptide matrix – SAPM) is able to inhibit demineralisation of the enamel by forming a layer on the tooth, like natural salivary pellicle, which buffers the acids and retains calcium phosphate during the demineralisation phase^26^. Such retained calcium phosphate may also facilitate extra remineralisation during the remineralisation phase.

*In vitro* data of SAPM have shown reduced demineralisation during acid attacks^26^. In addition, superior hardness recovery compared to bioglass, CPP-ACP, and fluoride varnish has been observed in a pH cycling model over 30 days^27^.

The aim of the present *in situ* clinical trial was to investigate the efficacy of a SAPM based gel developed for home care (Curodont PROTECT), in combination with daily oral hygiene, in remineralising artificial initial demineralisation and to compare its efficacy to daily oral hygiene alone, and the application of fluoride varnish (Duraphat, 22’600ppm Fluoride) and daily oral hygiene. Furthermore, the design of the present *in situ* trial allowed for the simulation of patients being at low caries risk (without brackets) and for high caries risk patients (with orthodontic brackets, respectively).

## Results

Nine volunteers participated in the study between 12/2014-07/2015 (7 female; mean age: 36.9 (18.6–58.4 years)). No breach of protocol by any participant was recorded and none were removed from analysis.

Stimulated salivary flow rate (CRT Paraffin Pellets, Ivoclar Vivadent, Liechtenstein) was within the normal range (average 3.6 ml/min at each examination). All volunteers lived in an area with up to 0.25 mg F^−^/L in the tap water, which has been constant for many years.

All possible randomised sequences of the cross-over design were used with a frequency as follows: 1xABC; 2xACB; 2xBAC; 1xBCA; 1xCAB; 2xCBA.

In total 162 specimens were inserted in the appliances. Five specimens fractured and could not be analysed (three specimens in the negative control group, one in the positive control group, one specimen in the test group). Hence, 157 specimens were available for statistical evaluation.

**Figure 1:**
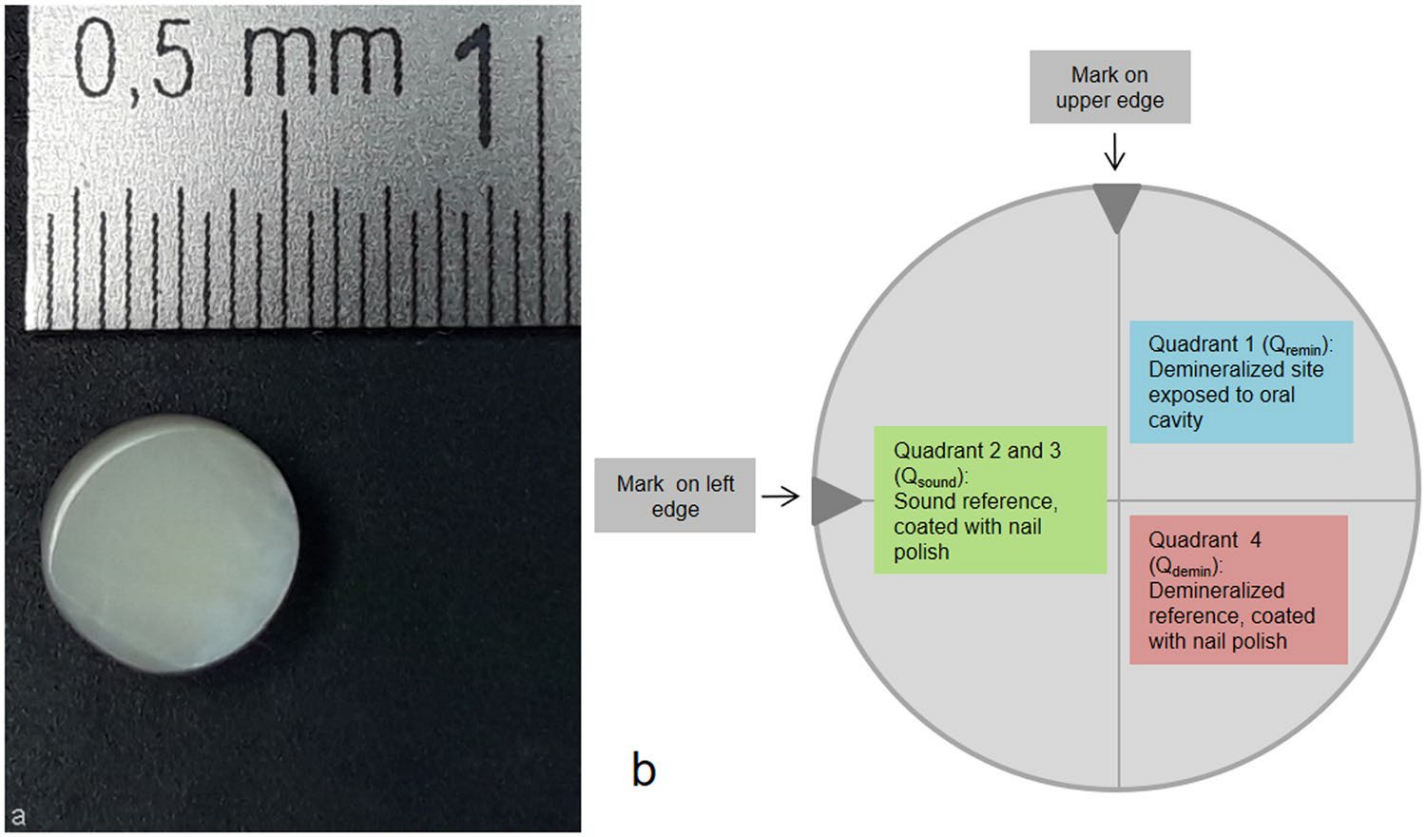
(**a**) Bovine specimen without bracket - before processing. (**b**) Schematic diagram of the various sample sections (sample without bracket).

Schematic diagrams of the various specimens are presented in Fig. 1 (sample without bracket) and 2 (sample with bracket).

### Laser fluorescence (LF) measurements

All specimens were classified as “caries free” prior to demineralisation (LF values 0–7, mean 1.6). After demineralisation, the fluorescence values of all specimens had increased, indicating “enamel lesions”. There were significant differences between the fluorescence values before and after demineralisation (Wilcoxon-Test, p < 0.0001). LF values and the resulting classification before and after the *in situ* phases are presented in Table 1.

**Table 1:** Laser fluorescence (LF) values of the study samples (standard deviation in parenthesis).

		Specimen without orthodontic brackets			Specimen with orthodontic brackets		
		Group A1 (Negative control)	Group B1 (positive control)	Group C1 (test group)	Group A2 (Negative control)	Group B2 (positive control)	Group C2 (test group)
Sound Enamel	LF values	2.04 (2.11)	1.74 (2.03)	1.70 (1.77)	1.23 (1.79)	2.59 (2.62)	1.07 (1.16)
	N (all category 0)	27	27	27	27	27	27
After Demineralisation	LF values	10.44 (2.60)	11.12 (3.76)	11.44 (2.85)	10.50 (2.02)	10.37 (2.17)	10.92 (3.30)
	N (all category 1)	25	26	27	26	27	26
After *in-situ* phase	LF values	15.72 (4.75)	11.96 (4.76)	8.56 (3.31)	16.27 (6.04)	11.67 (5.08)	7.7 (2.78)
	LF value change	+5.28	+0.85	−2.89	+5.77	+1.30	−3.15 (2.31)
	N category changes	decrease: 0 unchanged: 24 increase: 1	decrease: 3 unchanged: 22 increase: 1	decrease: 14 unchanged: 13 increase: 0	decrease: 0 unchanged: 22 increase: 4	decrease: 5 unchanged: 21 increase: 1	decrease: 16 unchanged: 10 increase: 0
Comparison of LF values: after demineralisation vs. after *in situ* phase		p = 0.0002	p = 0.088	p = 0.0002	p < 0.0001	p = 0.295	p < 0.0001
Comparison of LF categories: after demineralisation vs. after *in situ* phase		p = 0.0008	p = 0.547	p = 0.0008	p = 0.0005	p = 0.831	p < 0.0001
Comparison of LF values between groups after *in situ* phase		A1-B1: p = 0.0199; A1-C1: p < 0.0001; B1-C1: p = 0.0045			A2-B2: p = 0.0035; A2-C2: p < 0.0001; B2-C2: 0.001		

Laser Fluorescence categories: Category 0: 0–7, corresponds to sound enamel; Category 1: 8–24, corresponds to enamel lesions; Category 2: >24, corresponds to dentin lesions.

Negative control specimens without orthodontic brackets (A1) showed a significant increase in LF readings from 10.44 to 15.72 after the *in situ* phase (p = 0.0002) and in the categories (p = 0.008, Table 1). Negative control specimens with orthodontic brackets (A2) showed a significant increase in LF readings (10.50 to 16.27, p < 0.0001) and categories. Four specimens exhibited an increase of the category towards a deep lesion (Table 1).

Positive control specimens without (B1) and with orthodontic brackets (B2) remained almost unchanged in both LF readings and categories after the *in situ* phase. Test group specimens without (C1) and with orthodontic brackets (C2) both showed a significant decrease in LF readings and the corresponding categories after the *in situ* phase. LF readings for C1 specimens decreased from 11.44 to 8.56 (p = 0.0002) with 14/27 specimen corresponding to sound enamel. The LF values of C2 specimens decreased similarly from 10.92 to 7.70 (p < 0.0001) with 16/26 indicating sound enamel (Table 1).

In the specimens without orthodontic brackets (A1-C1) the LF readings of all three groups differed statistically (p < 0.0001). The pairwise comparison showed significantly lower LF values for C1 compared to both A1 and B1 after the *in situ* phase.

In the specimens with orthodontic brackets (A2-C2) the LF readings of all three groups differed statistically (p < 0.0001). The pairwise comparison (Table 1) showed significantly lower LF values for C2 after the *in situ* phase compared to both A2 and B2.

### Microcomputed tomography (micro-CT)

The micro-CT greyscale values of the specimens are described in Table 2 and Fig. 3. Due to time and cost constraints, as well as technical issues, randomly selected samples in each group were used for analysis by micro-CT, and the statistical analysis was adjusted accordingly. A post hoc power analysis of the micro-CT measurements was performed (G*Power, v 3.1). For the analysis of at least one sample in each group and each volunteer, a power of 0.8 for a large effect size (f = 0.37, α = 0.05) was calculated.

**Table 2:** Micro-CT greyscale values of the samples (standard deviation in parentheses).

	*Groups*	Without orthodontic brackets			With orthodontic brackets		
		*A1 (Negative control)*	*B1 (positive control)*	*C1 (test group)*	*A2 (Negative control)*	*B2 (positive control)*	*C2 (test group)*
Sound enamel	Q_sound_	115.57 (33.38)	121.07 (32.39)	115.37 (35.83)	130.25 (23.78)	122.19 (24.26)	111.55 (26.27)
After demineralisation	Q_demin_	100.21 (38.00)	93.54 (35.09)	103.22 (36.06)	115.30 (42.68)	115.78 (38.62)	93.53 (39.83)
After remineralisation	Q_remin_	111.26 (38.81)	106.40 (38.07)	122.04 (32.11)	117.98 (33.92)	126.55 (28.67)	146.78 (19.10)

**Figure 2:**
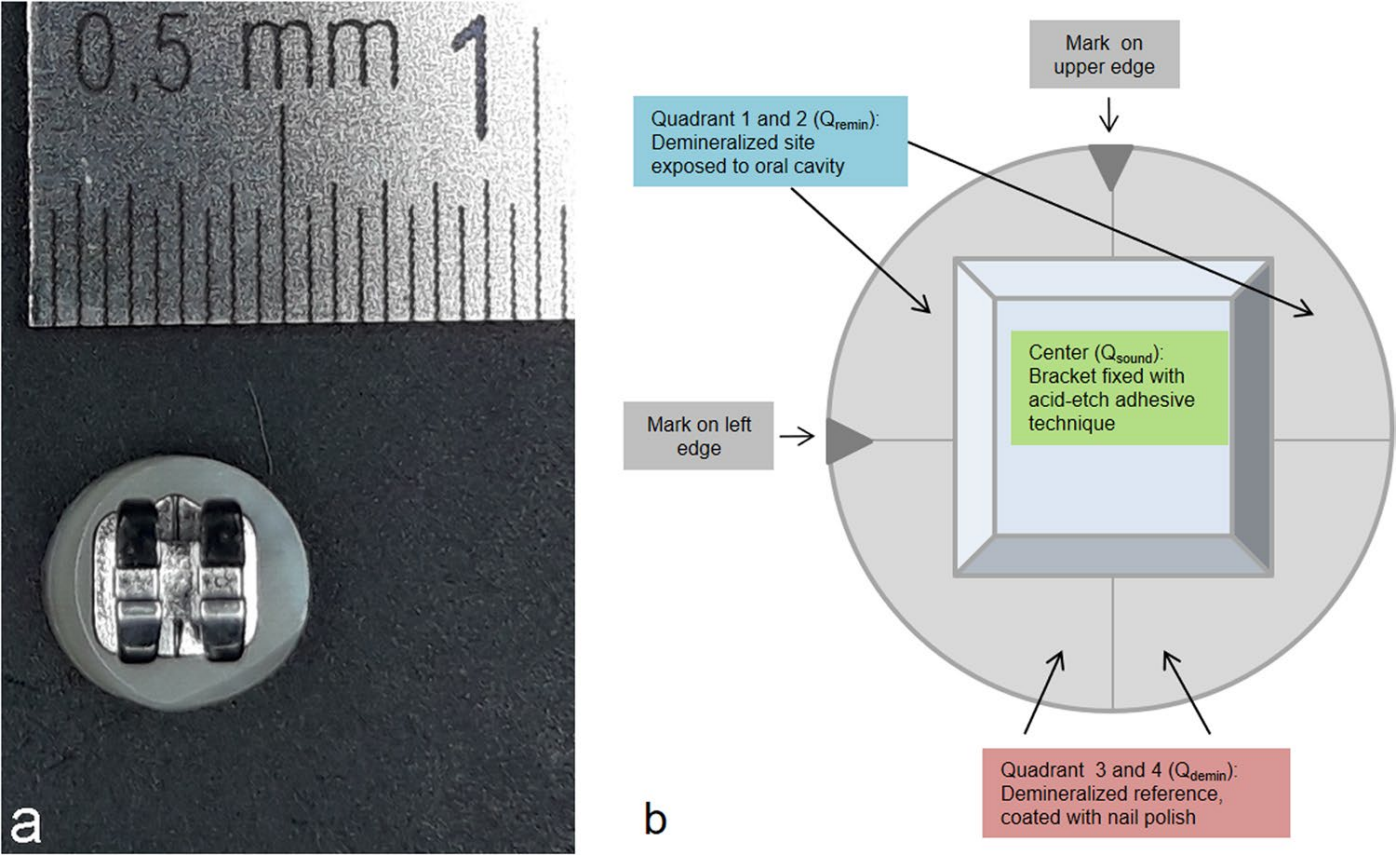
(**a**) Bovine specimen with bracket – before processing. (**b**) Schematic diagram of the various sample sections (sample with bracket).

**Figure 3:**
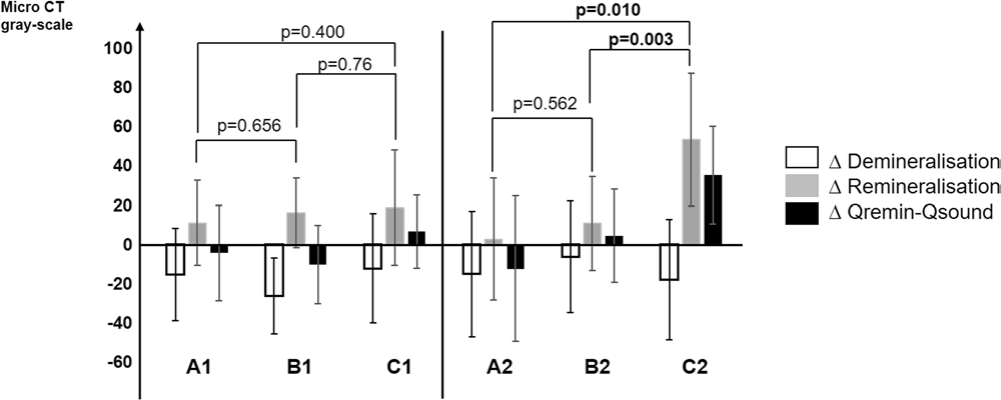
Δ of the greyscale values (mean values) for specimen without (A1, B1, C1) and with (A2, B2, C2) orthodontic brackets. Δ Demineralisation = greyscale values for the demineralised part of the specimens (ΔQ_demin_ − Q_sound_). D Remineralisation = greyscale values after exposure of the sample section in the oral cavity (ΔQ_remin_ − Q_demin_). ΔQ_remin_ − Q_sound_ = comparison of greyscale values between the sound enamel (untreated und not demineralised) with the sample section after exposure in the oral cavity; if positive, then gain of mineral compared to basic enamel, if negative, then loss of mineral compared to basic enamel.

Upon demineralisation, negative control specimens without orthodontic brackets (A1) showed a decrease in greyscale values from Q_sound_ = 115.57 to Q_demin_ = 100.21, followed by an increase during the *in situ* phase to Q_remin_ = 111.26, which is slightly below Q_sound_ (Table 2).

Positive control specimens without brackets (B1) showed an initial greyscale value of Q_sound_ = 121.07 decreasing to Q_demin_ = 93.54 after demineralisation and increasing to Q_remin_ = 106.40 during the *in situ* phase (Table 2).

Test group specimens without brackets (C1) showed an initial value of Q_sound_ = 115.37 decreasing to Q_demin_ = 103.22 after demineralisation, which increased to Q_remin_ = 122.04 during the *in situ* phase (Table 2), giving a higher mineral density of the enamel than before demineralisation (i.e. Q_sound_).

Negative control specimens with orthodontic brackets (A2) showed a decrease in greyscale values upon demineralisation from Q_sound_ = 130.25 to Q_demin_ = 115.30 followed by a slight increase after the *in situ* phase to Q_remin_ = 117.98 (Table 2).

Positive control specimens with brackets (B2) showed an initial value of Q_sound_ = 122.19 decreasing to Q_demin_ = 115.78 after demineralisation and again increasing to Q_remin_ = 126.55, indicating a higher mineral density than Q_sound_ (Table 2).

Test group specimens with brackets (C2) showed an initial value Q_sound_ = 111.55 decreasing to Q_demin_ = 93.53 upon demineralisation, and increasing to Q_remin_ = 146.78 after the *in situ* phase, indicating a higher mineral density than for the sound enamel control.

A comparison between the groups in the framework of multilevel models (Fig. 3) showed that specimens without brackets (A1-C1) did not yield a significant difference between the groups, although C1 specimens exhibited slightly higher greyscale values than the corresponding sound enamel control. For specimens with orthodontic brackets (A2-C2), the greyscale values of the test group (C2) were significantly different from both the negative control A2 (p = 0.01) and the positive control B2 (p = 0.003) after the *in situ* phase. For C2, Δ Q_remin_ − Q_sound_ values were positive, indicating substantially higher mineralisation.

## Discussion

The test group with SAPM exhibited both a significant decrease in LF and a significant increase in mineralisation as observed by micro-CT. SAPM has been shown previously to inhibit demineralisation and promote remineralisation during *in vitro* pH cycling models^26,27^. Yet, as the mineral density in the present *in situ* trial was higher after the remineralisation phase than in the sound enamel control (Fig. 3), additional minerals must have been incorporated in the specimens. This mineralisation might originate from a similar effect as that observed for monomeric SAP, where the formed SAPM was shown to bind minerals to form HA on the enamel surface^28^. SAPM has been shown to bind calcium phosphate from saliva and form a reservoir within its 3D structure, possibly increasing the availability of calcium and phosphate ions during the remineralisation phase^21,27^. In addition the immature bovine enamel specimens, which were obtained from calves, might have had mineralisation or maturation potential that matured human enamel does not display.

The orthodontic model was engaged to obtain faster progression of demineralisation in order to visualise changes within the 4-week wearing period^29^. For the test group, the orthodontic model (C2) showed substantially higher mineralisation by micro-CT after the *in situ* period than for the specimens without brackets (C1) after the *in situ* period. It should be noted that specimens with orthodontic brackets (A2-C2) were acid etched during the preparation in order to attach the brackets, possibly leading to lower mineralisation of the sound enamel surface under the brackets, which might have led to a steric hindrance to remove the SAPM from the specimen surface by normal oral functions as might have happened for C1.

It is unclear whether the clear difference in remineralisation seen in the micro-CT analysis among A2-C2 and its absence in the micro-CTs within A1-C1 is due to the additional handicap, or whether the larger standard deviation of the micro-CT measurements in contrast to the LF readings possibly masked clinical differences. Regardless, the significant decrease in LF and consequently the decrease in caries classification are in line with previous *in vitro* work on monomeric P11-4^30^.

Studies are available regarding the ability to use laser fluorescence devices (DIAGNOdent and DIAGNOdent pen) for measurements of demineralisation and remineralisation in laboratory-based and clinical studies^30–32^. In a recent clinical study, the additional effect of the application of monomeric SAP was compared to the application of fluoride varnish alone in active occlusal initial caries lesions on erupting permanent molars. After three and six months, the test group showed significantly superior lesion regression compared to the control group, both in the laser fluorescence readings and in the clinical assessment of the caries stage and activity^33^.

The laser fluorescence measurements may be dependent on the angle of the device with the specimen’s surface, as it is used freehand in clinical practice. To minimise such error, the tip B was used in our study, which is flat and recommended for use on smooth surfaces to avoid any effect on the measurements.

This is the first study using a SAPM based gel (+containing fluorides) developed for home care (Curodont PROTECT) and comparing the caries preventive effect with the established treatment using fluoride varnish. Clinical proof of the prevention of carious lesions is difficult, as it typically requires large patient populations to be followed over a lengthy period of time. An alternative for near-clinical data is the use of *in situ* models^34^, in which volunteers wear enamel specimens for a defined period of time and the specimens are then available for detailed analysis of the progression of caries with techniques not available in clinical testing, such as micro-CT. Yet the *in situ* models do not come without compromises and limits. Firstly, the wear time needs to be limited to a few months. Secondly, the enamel specimens are either directly bonded to the teeth of the volunteer and caries lesions might possibly occur around it, or they are included in an appliance to be worn by the volunteer, providing the opportunity to do a sequential or cross-over study design. Yet if worn in an appliance the specimens together with the appliance are removed during meals, thus reducing exposure to natural demineralisation risk. Lastly, the appliance is cleaned separately from the teeth and might not get the same oral hygiene as the volunteer’s dentition.

In the present study the specimens with and without brackets were not assigned randomly to the left and right side of the appliances. As oral hygiene is known to differ for the right and left side due to the habit and handedness of the patient, bias might have been introduced, since the specimens with brackets were all situated at one site in all patients. Nevertheless, the appliances were cleaned separately outside of the mouth and hence no significant influence of either habits or handedness might have occurred.

The study design consisted of three arms of four weeks each, with a one-week washout phase between them. *In situ* remineralisation models already led to the observation that the greatest remineralisation took place after one week and remained constant for two to three weeks thereafter^35^. Other research groups have shown that the effects of various fluoride concentrations on enamel samples were already measurable after two weeks^36^. A duration of four weeks was chosen for the present study, as it was not known how long the SAPM would take to show its efficacy in the *in situ* model. The one-week washout period is in line with previous studies^37^ and was chosen to ensure that all traces of treatment products were cleared out of the oral cavity. It should be noted that all treatments were applied extra-orally in order to minimise cross contamination.

Due to hygienic and ethical considerations, *in situ* studies are often conducted with bovine specimens. They are widely used in enamel and caries research, but are by no means identical to human teeth. Bovine enamel is initially covered with a cementum layer which is removed by grinding, leaving a prismatic structure on the surface, quite unlike the natural aprismatic, fluoride-rich, matured enamel surface of human teeth^38^. Yet a natural prismatic surface is formed throughout the *in situ* period.

The bovine enamel specimens used in the present study show the expected natural variation of mineralisation for all groups. The chemical demineralisation led to fairly homogeneous artificial caries lesions as observed by LF and micro-CT. The negative control group (A1, A2) showed a slight increase in the laser fluorescence values, and a slight increase in mineralisation identified by micro-CT. These at first glance contradictory findings can be explained by the fact that enamel samples will accumulate bacterial porphyrins during the *in situ* phase, increasing the LF value and more than compensating for the remineralisation effect. The positive control (B1, B2) showed unchanged LF values and an increase in mineralisation. Because fluoride is incorporated in the top layer of the enamel lesion, fewer porphyrins would penetrate the enamel than in the negative control.

## Conclusion

The study shows that the SAPM has a clinical beneficial effect in caries prevention, as has been previously shown *in vitro*^26,27^. Furthermore, the use of the home care SAPM-based product proved to be useful in preventing the progression of initial demineralisation, especially around orthodontic brackets. In the present study a concentrated SAPM product was used twice a week. Nevertheless, the efficacy of such a product depends on patient’s compliance and, if possible, SAPM could be incorporated into toothpastes for daily use. Regardless, the SAPM-based product (also containing fluorides) demonstrated superior remineralisation potential compared to the present gold standard of fluoride varnish alone. The *in situ* model used in this study has its shortcomings, and clinical trials should follow to verify the present results.

In the absence of a prospective, randomised clinical trial and within the limitations of the *in situ* model, SAPM showed its potential for prevention of demineralisation and remineralisation of enamel, especially in high caries risk patients, where fluoride alone might not be sufficient, such as patients with fixed orthodontic appliances.

## Materials and Methods

### Study design and patient population

This randomised, crossover *in situ* clinical trial was performed according to the Declaration of Helsinki and in compliance with ISO 14155:2012. The study was approved by the Ethics Committee of the Medical Faculty of the Philipps-University of Marburg, Germany (Code 33/14, date of approval: 7 April 2014) and registered in the German Clinical Trials Register (DRKS00006215, date of registry: 4 June 2014).

Nine volunteers were recruited at a dental practice (Lollar, Germany), and written informed consent was obtained prior to any study-related procedures. Volunteers needed to be able and willing to observe good oral hygiene and follow the instructions given. In detail, the inclusion criteria were: age ≥18 years, low caries activity, participants willing and able to attend the on-study visits and assessments, participants willing and able to understand all study-related procedures and to follow the self-treatment instructions, and informed consent.

The exclusion criteria were: subjects with removable partial denture, subjects with fixed orthodontic appliances, subjects with current dental trauma or surgery, recently (<2 weeks) applied high-concentration fluoride treatment (elmex gelee, etc.), smokers, subjects with bronchial asthma, evidence of tooth erosion, a history of head and neck illnesses (e.g., head/neck cancer), pregnant and breast-feeding women, any pathology or concomitant medication affecting salivary flow or dry mouth (unstimulated <0.2 ml/min), last utilisation of antibiotics <2 months, patients receiving medication known to stain teeth, such as tetracycline or chlorhexidine, a high caries risk, concurrent participation in another clinical trial, subjects with known allergies//hypersensitivity towards ingredients in Curodont Protector of Duraphat varnish, respectively.

No dental treatment was performed throughout the study period.

### Enamel specimens

Extracted bovine incisors were used in this study^39^. All teeth were examined using a stereomicroscope (Leica MS 5, Leitz, Wetzlar, Germany) at x16 and x25 magnification to determine the presence or absence of enamel cracks or discolouration. The bulk of the adherent soft tissues was carefully removed using scalpels, and teeth were stored in 0.9% NaCl. Up to three specimens were prepared from one incisor under running water using a trephine bur (5 mm diameter; Komet, Lemgo, Germany). The dentin surface of the resulting specimens was ground to give a plane parallel specimen of ~2 mm thickness. The enamel surfaces of the specimens were ground and polished progressively up to 4,000 grit (polishing paper: Hermes, Hamburg, Germany), aiming at the removal of the natural bovine cementum layer. The resulting specimens’ enamel surfaces were examined again microscopically to ensure the absence of defects or physical damage. Specimens without obvious signs of cracks or discolouration were included in the study (n = 162). All specimens were sterilised by ultrasonication for 2 min in 2% sodium hypochlorite followed by ultrasonication in 70% ethanol for another 2 min. Following this, the samples were washed twice in sterile distilled water^40^ and stored until further use in dH_2_O.

### Preparation of enamel specimens without orthodontic brackets

One-half of each specimens’ surface was covered with clear, acid-resistant nail polish (Manhattan, Mainz, Germany) yielding an internal sound enamel control (Fig. 1, Quadrants 2 and 3: Q_sound_). Specimens were demineralised for 20 days in a demineralisation solution in an incubator (Type B 290, Heraeus GmbH, Hanau, Germany) at a constant temperature of 37 °C. The enamel lesions were prepared by immersion in 5 litres of a solution containing 6 µM MHDP, 3 mM CaCl_2_ × 2 H_2_O, 3 mM KH_2_PO_4_ and 50 mM CH_3_COOH^34,41^. The set pH = 4.95 (4.92–4.98) was measured daily (HI 98127, HANNA Instrument, Kehl am Rhein, Germany) and adjusted by the addition of HCl when needed to maintain a constant pH = 4.92–4.98 throughout the demineralisation period. Demineralisation was stopped when Laser Fluorescence (LF) (DIAGNOdent, KaVo, Biberach, Germany) values corresponded to the values of initial caries lesions^42^, and samples were examined microscopically to check for a dull, whitish surface resembling typical early carious changes of enamel and the absence of cavitation. All specimens were stored in sterilised distilled H_2_O at constant temperature (37 °C) (Incubator Type B 290, Heraeus GmbH, Hanau, Germany). Half of the demineralised tooth surface was covered with nail polish, yielding an internal demineralised control (Fig. 1, Quadrant 4, Q_demin_). The remaining quadrant (Quadrant 1, (Q_remin_) was thus the demineralised surface exposed to the oral cavity throughout the *in situ* phase.

### Preparation of enamel specimens with orthodontic brackets

After etching the enamel surface with 36% phosphoric acid gel for 15 seconds (Conditioner 36, Dentsply DeTrey, Konstanz, Germany) the surface of the enamel was rinsed with clean and oil-free water spray and dried using the three-in-one-syringe. Then metal mini-orthodontic brackets (3 × 3 mm, SPEED System Orthodontics, Canada) were attached following manufacturer’s instructions (Transbond XT primer and adhesive, 3M Unitek, Landsberg, Germany) in the centre of the specimen^29^ and light cured for 10 seconds (Bluephase, Ivoclar Vivadent AG, Schaan, Liechtenstein). Hence, the specimen’s surface below the bracket served as an internal sound enamel control (Fig. 2, centre of the samples: Q_sound_). Specimens were then demineralised as described above and half covered with nail polish (Fig. 2, Quadrants 3 and 4, Q_demin_) yielding an internal demineralised control. The remaining uncovered surface (Fig. 2, Q_remin_) thus formed the demineralised surface exposed to the oral cavity throughout the *in situ* phase.

### Preparation of appliances

Each volunteer’s mandible impression was taken and individual removable acrylic appliances (thickness 0.5 mm) were prepared (Erkodur clear, Erkodent, Pfalzgrafenweiler, Germany). Three specimens were fixed on each buccal flange of the appliance (Palapress, Heraeus Kulzer, Hanau, Germany). The specimens with orthodontic brackets were placed on the left side and the specimens without orthodontic brackets were placed on the right side of the appliance. Care was taken to avoid maxillary teeth coming in contact with the specimens.

### Treatment during *in situ* phase

This crossover *in situ* study was divided into three phases. Each phase lasted four weeks followed by a one-week washout period^37^ to avoid any carry-over effects of the treatments. The groups and respective treatments were: (A) negative control, no treatment; (B) positive control, fluoride varnish (Duraphat, containing 22,600 ppm fluoride; Colgate-Palmolive, New York, USA); Application: 1x in-office at the start; C: test group, SAPM (Curodont PROTECT, containing 1000 ppm P11-4 and 900 ppm fluoride; Credentis AG, Windisch, Switzerland); Application: first application in-office at start by investigator/study team, followed by twice-weekly applications by the volunteer at home (twice-weekly SMS reminders were sent to volunteers; the remainder of the treatment product was collected to check for protocol adherence). Volunteers were randomly allocated to a treatment sequence. A randomisation list was generated prior to the study by a statistician with all possible sequences. The list with the random numbers and the resulting sequences were centrally stored. The principle investigator was notified by telephone of the sequence for each new patient. The following sequences were possible and the order was generated randomly by a statistician: ABC, ACB, BAC, BCA, CAB, CBA.

During each washout phase, volunteers did not wear the appliance, nor did they use any of the products under investigation. The appliances were collected by the investigator and an appliance with six new specimens was handed out for the next treatment phase.

Volunteers were instructed to brush their teeth twice daily without the appliances. A toothbrush (Oral B Indicator, Procter and Gamble, Schwalbach am Taunus, Germany) and fluoride toothpaste (blend-a med classic, 1450ppm sodium fluoride, Procter and Gamble, Schwalbach am Taunus, Germany) were provided for each treatment phase. Appliances were to be cleaned separately with the same toothbrush and toothpaste. No other dental products (mouth rinse, gels, etc.) except dental floss were to be used throughout the study. Appliances were worn 24 hours a day, to be removed only while eating or tooth brushing, during which time they were stored in a little jar provided to keep them moist.

Volunteers were instructed to follow their normal eating habits throughout the study as far as possible. At the end of a treatment phase, all specimens were removed from the appliance, rinsed with dH_2_O and disinfected. Brackets (and anything stuck to them) were carefully removed, as they would have resulted in disturbances of the micro-CT analysis.

### Laser fluorescence (LF) measurements

The specimens’ LF values (DIAGNOdent 2095, KaVo, Biberach, Germany) were recorded before and after demineralisation and after the *in situ* period. Assessments were performed with a calibrated device using Probe Tip B for smooth surfaces, and the appropriate function-set. Specimens were dried (5 sec), and measurements were performed, recorded and categorised^42^: values from 0–7 corresponded to sound enamel surfaces, values from 8–24 corresponded to enamel lesions and values >24 corresponded to dentin lesions.

### Micro-computed tomography (micro-CT)

**Figure 4:**
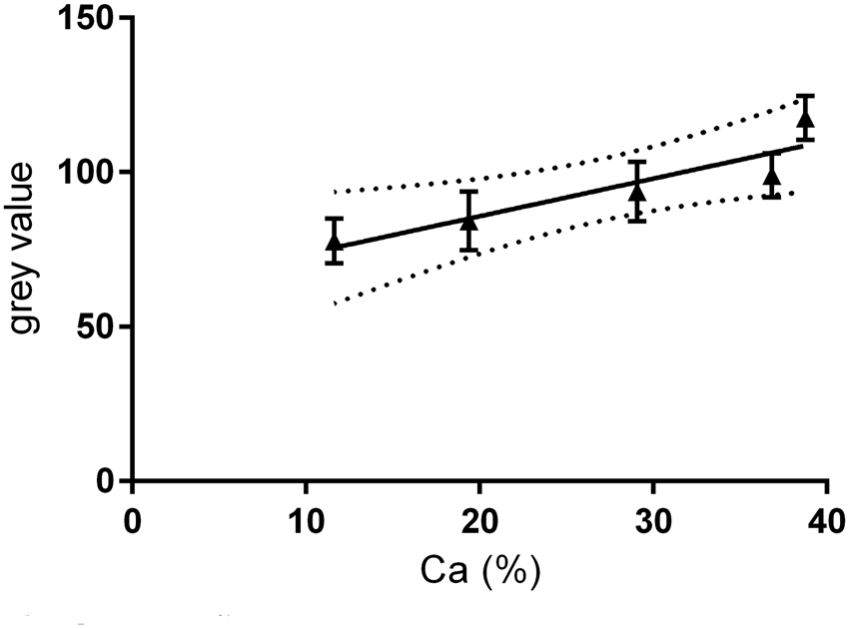
Calibration curve (8-bit grey value versus mineral content %Ca).

**Figure 5:**
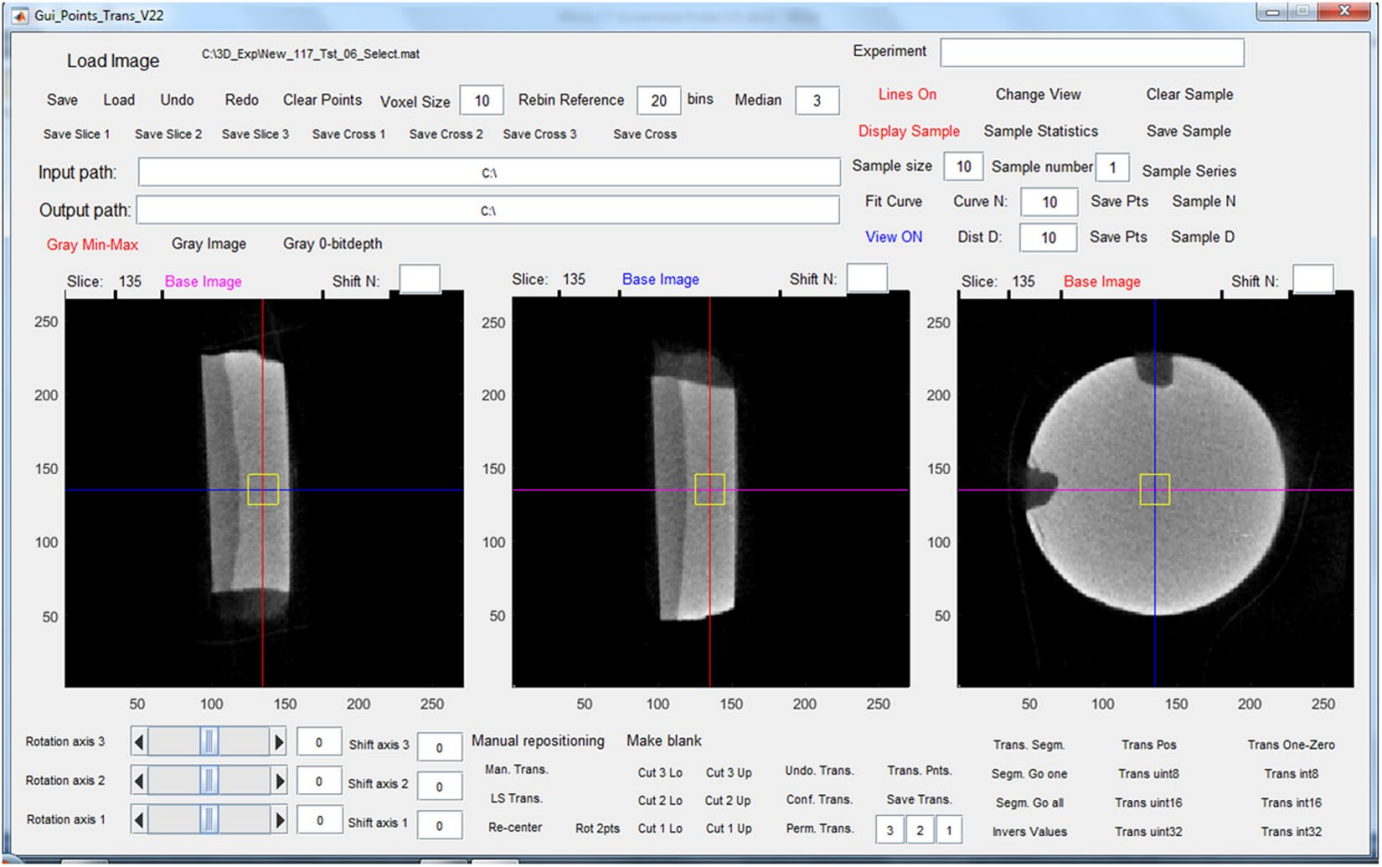
Screenshot of a micro-CT scan showing the graphical user interface. The interface shows the sample in three sectioning planes. The region of interest (yellow square in screenshot) can be chosen at different locations, here as example placed centrally in the sample.

Specimens were scanned with the micro-CT SCAN Skyscan 1172 (Bruker, Kontich, Belgium). The sensor was set to the maximum resolution of 6.75 µm voxel size in a field of view of 4000 by 2664 pixels, and specimens were scanned with a tube voltage of 84 kV and a current of 118 µA for an exposure time of 6450 ms. A ring artefact correction was used. Calibration curves of the micro-CT images were obtained with the aid of a hydroxyapatite phantom containing five hydroxyapatite/hydroxyethylcellulose samples of differing yet known density. The relationship between greyscale values and calcium content was approximately linear (R^2^ = 0.8424, p = 0.03) (Fig. 4), and thus the grey values measured correlated with the mineral content of the specimens’ volume of interest^43^. The digitised images were captured and analysed using customised software (Gui-Points22) written in Matlab (Matlab, The MathWorks, Natick, Massachusetts, USA). In each specimen, three ranges were first identified for each region of interest (ROI), that is, Q_sound_, Q_demin_ and Q_remin_ and the grey-value profile defined for each investigation site. The range of grey-value quantifications was determined on cells of 10 × 10 × 10 voxels for each ROI (Fig. 5).

### Statistical analysis

The required sample size was ascertained on the basis of previous results of comparable study designs^44,45^. Sample size calculation was run with PASS 2008 software (inequality tests and non-inferiority tests for mean values, based on differences). A power of 90% for nine volunteers with six plates each was calculated.

Anonymised key lists were kept by the principle investigator. Data privacy laws and doctor-patient confidentiality were observed at all times.

Assessor blinded analysis was performed on all specimens, and unblinding was done afterwards. Statistical evaluation was performed using IBM SPSS® Statistics (Version 23) and MedCalc (Version 17.9.7). The data were subjected to Shapiro-Wilk’s test to check for normality and were found not to be normally distributed. Hence, non-parametric tests were used for statistical analysis. The Friedman test was used to compare the LF readings between the groups. The Wilcoxon test was used to analyse significant differences between the LF measurements at different times (before/after demineralisation, after the *in situ* phase) and for pairwise comparison between the groups.

The experimental design resulted in a clustered data structure, as specimens worn by the same volunteer were exposed to the same influences (such as dietary habits, dental hygiene, composition of the saliva, etc.). In order to take account of this data structure, linear mixed models were calculated and significance tests were performed for differences within the scope of multilevel models.

The significance level was set at α = 0.05.

## Data Availability

The datasets generated during and analysed during the current study are available from the corresponding author on request.
